# The association between smoking exposure and endothelial function evaluated using flow-mediated dilation values: a meta-analysis

**DOI:** 10.1186/s12872-024-03915-x

**Published:** 2024-06-05

**Authors:** Xiaoxiao Jia, Peng Zhang, Liping Meng, Weiliang Tang, Fang Peng

**Affiliations:** 1 Department of Pathology, Shaoxing People’s Hospital, Shaoxing, China; 2 Department of Cardiology, Shaoxing People’s Hospital, Shaoxing, China

**Keywords:** Endothelial function, Flow mediated dilatation, Smoke, meta-analysis

## Abstract

**Background:**

Tobacco use is recognized as a major cause of cardiovascular disease, which is associated with endothelial dysfunction. Endothelial function is evaluated using flow-mediated dilation (FMD), which is a noninvasive method. This meta-analysis aimed to investigate the association between smoking exposure and endothelial function evaluated using FMD values.

**Methods:**

We searched the PubMed, Embase, Web of Science, and Cochrane Library databases for cohort studies of smokers or passive smokers that used FMD to assess endothelial function. The primary outcome of the study was the change in the rate of FMD. The risk of bias was evaluated using the Cochrane Collaboration tool and Newcastle–Ottawa Scale. Further, the weighted mean difference was used to analyze the continuous data.

**Results:**

Overall, 14 of 1426 articles were included in this study. The results of these articles indicated that smoking is a major cause of endothelial dysfunction and altered FMD; a pooled effect size of − 3.15 was obtained with a 95% confidence interval of (− 3.84, − 2.46). Notably, pregnancy status, Asian ethnicity, or health status did not affect heterogeneity.

**Conclusions:**

We found that smoking has a significant negative impact on FMD, and measures such as medication or education for smoking cessation may improve endothelial function and reduce the risk of cardiovascular disease.

**Trial Registration:**

The meta-analysis was registered with PROSPERO on April 5th, 2023 (CRD42023414654).

**Supplementary Information:**

The online version contains supplementary material available at 10.1186/s12872-024-03915-x.

## Background

Tobacco-related diseases are recognized as a significant global burden, causing an estimated 7 million deaths per year. Moreover, tobacco and tobacco smoke contain over 7,000 chemicals, most of which are toxic [1]. Compared to nonsmokers, smokers have a 20-year shorter life expectancy, resulting in significant economic implications [2, 3]. Moreover, smoking is well-known as a risk factor for the development and progression of cardiovascular diseases (CVDs), such as ischemic stroke, coronary artery disease, and peripheral artery disease. Approximately 10% of all cardiovascular deaths in adults are caused by cigarette smoking [4].

Cardiovascular morbidity and mortality due to smoking can be attributed to various pathophysiological mechanisms [5]. Notably, endothelial dysfunction is considered a crucial early sign of the development of coronary atherosclerosis, and its early detection may help prevent CVDs. Several studies, both clinical and animal-based, have reported that exposure to cigarette smoke and its components can induce vascular endothelial pathology by reducing the availability of nitric oxide (NO). The reaction of NO with free radicals present in smoke together with direct physical damage to endothelial cells results in altered biosynthesis and reduced activity of NO [6, 7]. Consequently, the ability of the endothelium to maintain its vasodilatory and anti-inflammatory, -thrombotic, and -oxidant effects is impaired.

Assessment of endothelial function is usually performed using flow-mediated dilation (FMD), which is a noninvasive approach for measuring changes in the diameter of the brachial artery in response to shear stress induced by reactive hyperemia [8]. In the present study, we performed a systematic review and meta-analysis to evaluate the association between smoking exposure and endothelial function evaluated using FMD values. Furthermore, the effects of factors such as pregnancy status, Asian ethnicity, and health status of smokers on endothelial function were examined.

## Methods

We followed the statements on the Preferred Reporting Items for Systematic Reviews and Meta-analyses and the Meta-analysis of Observational Studies in Epidemiology. The meta-analysis was registered with PROSPERO on April 5th, 2023 (CRD42023414654).

### Search strategy

A comprehensive literature search in the PubMed, EMBASE, Cochrane Library, and Web of Science databases was conducted. The date limit was invalid until February 21, 2023. Searches included index and text terms and were limited to titles or abstracts (see the Additional file 1 for the complete search strategy). No language or study design restrictions were employed. The search strategy was adapted to the syntax applicable to each database.

### Inclusion and exclusion criteria

The inclusion criteria were as follows: studies published in English or Chinese, studies on smokers or passive smokers, studies assessing endothelial function using FMD, and observational studies. The exclusion criteria were as follows: abstracts, letters, talks, or reviews; studies in which FMD was not used to assess endothelial function; studies involving animal testing; studies with insufficient data for statistical analysis; studies without nonexposure group; studies whose full text was not available; and duplicate studies with data already included in the meta-analysis.

### Data extraction

All included studies were independently examined by three reviewers, and the following data were extracted: author; publication year; study design type; region of data set; cigarette type; N, mean, and standard deviation of the experimental and control groups; inclusion of patient characteristics, including pregnancy status, health status, and sex. Should disagreements occur, a fourth reviewer will be consulted.

### Quality assessment

Three reviewers independently scored the quality of the included studies using the Newcastle–Ottawa Scale (NOS) criteria, which can provide a maximum score of 9. This scale evaluates the quality of non-randomized, cohort, and case-control studies in relation to their design, content, and ease of use. When in doubt or should discrepancies occur, a fourth researcher will be consulted.

### Statistical analysis methods

Heterogeneity was detected using Q-test and I^2^-test, and a random-effects model was selected if I^2^ was greater than 50%. A subgroup analysis was conducted to assess sources of heterogeneity between studies. The following subgroups were considered: tobacco type, pregnancy status, sex, and the region to which the exposed population belonged. Continuous data were combined using weighted mean difference statistics. Statistical significance was set at a p-value less than or equal to 0.05, and STATA 15.1 was used for all statistical analyses.

## Results

### Basic characteristics

**Fig. 1:**
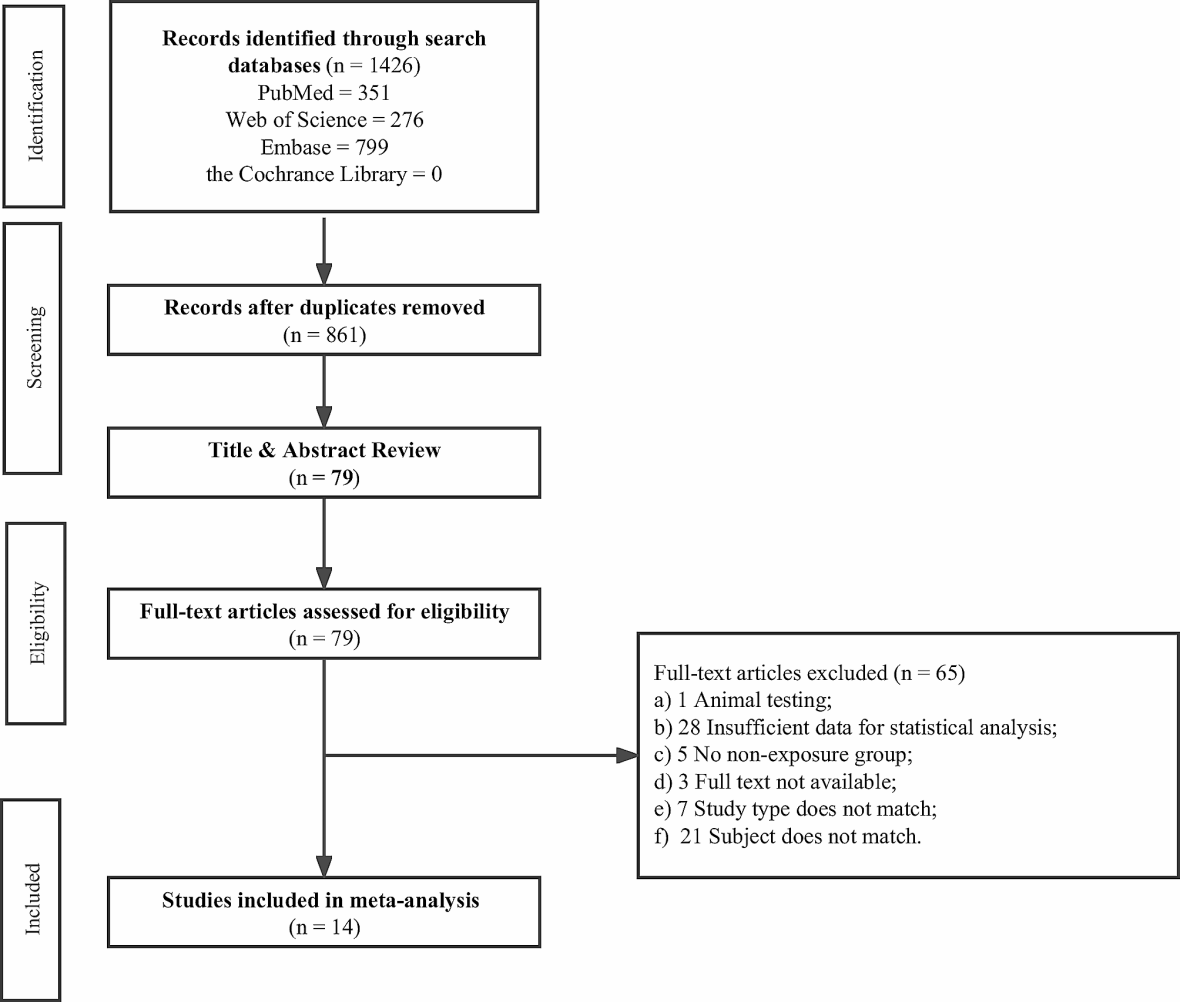
Flowchart of the selection of eligible studies

The flowchart for the selection of eligible studies is shown in Fig. 1. The database search identified 1426 publications. The detailed search strategy is presented in Additional file 1. The titles and abstracts of the remaining 861 publications were reviewed after eliminating duplicate records. After a thorough review, 79 of these publications were deemed eligible. After reviewing the full text of 79 articles, 65 of these publications did not satisfy our inclusion criteria. Specifically, one was an animal trial, 28 had insufficient data for statistical analysis, five did not have a nonexposure group, three were not available in full text, seven did not match the study type, and 21 did not match the topic. Finally, 14 articles were determined to be eligible for inclusion in this meta-analysis.

The NOS scores of the included studies are reported in Table 1. Most studies were of good quality, with quality assessment scores of ≥ 7 (out of 9), and only one had a score of 5 (Table 1).

**Table 1:** Results of quality assessment using the Newcastle-Ottawa Scale for observational studies

Study	Year	SELECTION				COMPARABILITY	EXPOSURE			Score
		Representativeness of the exposed cohort	Selection of non-exposed cohort	Assessment of exposure	Identify outcome not yet available for observation at study entry	The comparability of the exposed and unexposed	Assessment of outcomes	Was follow-up long enough for outcome to occur	Non-Response Rate	
Nicolau	2011	★	★	★	★	★	★	★	★	8
Heffernan	2010	★	★		★	★★	★	★	★	8
Corretti	1998	★	★	★	★	★★	★	★	★	9
Hashimoto	2021	★	★		★	★	★			5
Miyata	2015	★	★		★	★	★	★	★	7
Suzuki	2020	★	★		★	★	★	★	★	7
Ozaki	2010	★	★		★	★	★	★	★	7
Esen	2004	★	★	★	★	★	★	★	★	8
Quinton	2008	★	★		★	★	★	★	★	7
Tanriverdi	2006	★	★	★	★	★	★	★	★	8
Gaber	2016	★	★		★	★	★	★	★	7
Diab	2015	★	★	★	★	★	★	★	★	8
Yao	2011	★	★	★	★	★★	★	★	★	9
Ludwig	2010	★	★		★	★★	★	★	★	8

### Meta-analysis results

An overview of the characteristics of the included studies is provided in Table 2 [9–22]. Overall, there were 17 observational studies published from 1998 to 2021, and tobacco types were classified into two categories: conventional and hookah. Four studies compared outcomes between pregnant and nonpregnant women, nine studies mentioned sex of the participants, and four studies included unhealthy populations.

**Table 2:** Characteristics of the included studies

Author	Year	Study design	Region	Gender	Pregnant	Health status	Cigar type	Exposed group			Non-exposed group		
								*N*	Mean	Sd	*N*	Mean	Sd
Nicolau [9]	2011	Observation	Brazil	Female	Non-pregnant	Healthy woman	Cigarettes	19	7.21	5.57	34	10.52	4.76
Nicolau [9]	2011	Observation	Brazil	Female	Pregnant	Pregnant	Cigarettes	33	8.74	4.83	47	11.5	5.77
Heffernan [10]	2010	Observation	America	Female & Male	-	Chest pain	Cigarettes	26	8.9	0.9	39	12.6	0.7
Corretti [11]	1998	Observation	America	Male	-	Coronary artery (CAD)	Cigarettes	12	1.9	7.4	13	11.4	7.2
Hashimoto [12]	2021	Observation	Japan	Male	-	Healthy	Cigarettes	331	4.9	2.7	1181	6.6	3.4
Miyata [13]	2015	Observation	Japan	-	-	Healthy	Cigarettes	18	8.7	4	14	8.5	4.8
Suzuki [14]	2020	Observation	Japan	Female	-	Healthy	Cigarettes	10	5.07	1.79	10	7.92	3.01
Ozaki [15]	2010	Observation	Japan	Male	-	Healthy man	Cigarettes	9	5	2.6	11	9.5	5.2
Esen [16]	2004	Observation	Turkey	Female & Male	-	Healthy	Cigarettes	20	4.7	1.6	20	9.2	4.6
Quinton [17]	2008	Observation	Australia	Female	Pregnant	Pregnant	Cigarettes	21	4	2.3	20	9.7	4
Tanriverdi [18]	2006	Observation	Turkey	Female & Male	-	Coronary arteries	Cigarettes	36	5.7	2.2	51	6.81	1.95
Gaber [19]	2016	Observation	Egypt	Male	-	Healthy man	Water pipes	10	4.8	0.5	20	8.7	0.6
Gaber [19]	2016	Observation	Egypt	Male	-	Healthy man	Cigarettes	10	5.1	0.6	20	8	0.3
Diab [20]	2015	Observation	Egypt	Male	-	Healthy man	Water pipes	30	12	5.94	17	17.45	11.29
Diab [20]	2015	Observation	Egypt	Male	-	Healthy man	Cigarettes	30	12.19	7.42	17	17.45	11.29
Yao [21]	2011	Observation	China	Male	-	Erectile dysfunction	Cigarettes	21	6	0.8	48	9.7	2.5
Ludwig [22]	2010	Observation	Germany	Female	Non-pregnant	Healthy woman	Cigarettes	17	8.5	3.7	15	9.9	4.5

-: Not mentioned in the article

**Fig. 2:**
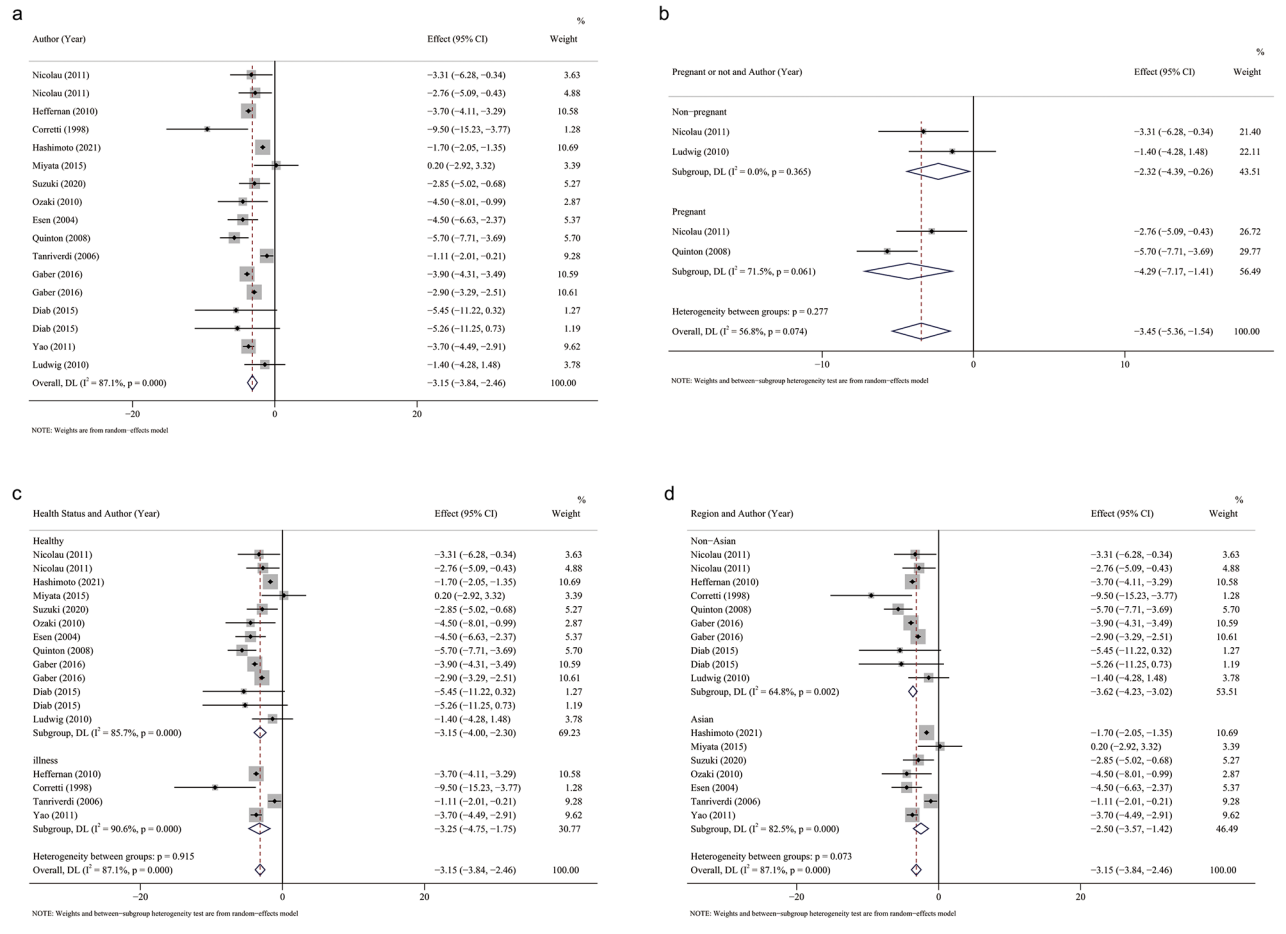
**a**: Forest plot of the included studies. **b**: Forest plots for determining whether the included population is pregnant. **c**: Forest plot of the health status of the subgroup analysis. **d**: Forest plot of regions of the subgroup analysis

The pooled effect size was − 3.15 with a 95% confidence interval of (− 3.84, − 2.46), and the results were statistically significant (*P* < 0.01). The results showed that smokers had reduced FMD values compared to non-smokers, while reduced FMD values indicated impaired vascular endothelial function. Therefore, smoking may affect the vascular endothelial function of patients and increase the risk of cardiovascular disease (Fig. 2a).

In terms of age, there was no significant difference in the age distribution between the exposed and non-exposed groups (*P* = 0.9076). As for the gender ratio, it could not be statistically analyzed because there were only three articles in the study that mentioned the gender ratio (Table 3).

**Table 3:** Age Distribution of Exposed and Non-Exposed Groups Go to Situation

Author	Year	Exposed group			Non-exposed group			*P*
		**Age Mean**	**Age Sd**	**Female (%)**	**Age mean**	**Age sd**	**Female (%)**	0.9076
Nicolau [9]	2011	26.03	2.88	100	25.28	2.77	100	
Nicolau [9]	2011	24.54	3.13	100	25.33	3.25	100	
Heffernan [10]	2010	58	2	59	58	2	62	
Corretti [11]	1998	47	9	0	46	7	0	
Hashimoto [12]	2021	58	12	0	47	16	0	
Miyata [13]	2015	21	1	/	20	1	/	
Suzuki [14]	2020	21	1	100	21	1	90	
Ozaki [15]	2010	30	4	0	27	3	0	
Esen [16]	2004	27	9	75	25	7	70	
Quinton [17]	2008	26.6	5.8	100	27.5	5.1	100	
Tanriverdi [18]	2006	52.8	10.7	41	56.1	10.5	39	
Gaber [19]	2016	/	/	0	/	/	0	
Gaber [19]	2016	/	/	0	/	/	0	
Diab [20]	2015	35.16	10.91	0	35.52	10.33	0	
Diab [20]	2015	35.16	10.91	0	35.52	10.33	0	
Yao [21]	2011	31.6	6.0	0	32.5	7.3	0	
Ludwig [22]	2010	32.4	2.9	100	29.7	2.9	100	

/: Not mentioned in the article

### Subgroup analysis

Subgroup analysis demonstrated that pregnant and nonpregnant populations were not a source of heterogeneity. Smoking alone reduced FMD regardless of pregnancy (Fig. 2b). Similarly, a good or bad health status was not a cause of heterogeneity (Fig. 2c). Finally, the presence or absence of Asian populations was also not a source of heterogeneity, suggesting the existence of other unknown potential causes of heterogeneity (Fig. 2d).

**Fig. 3:**
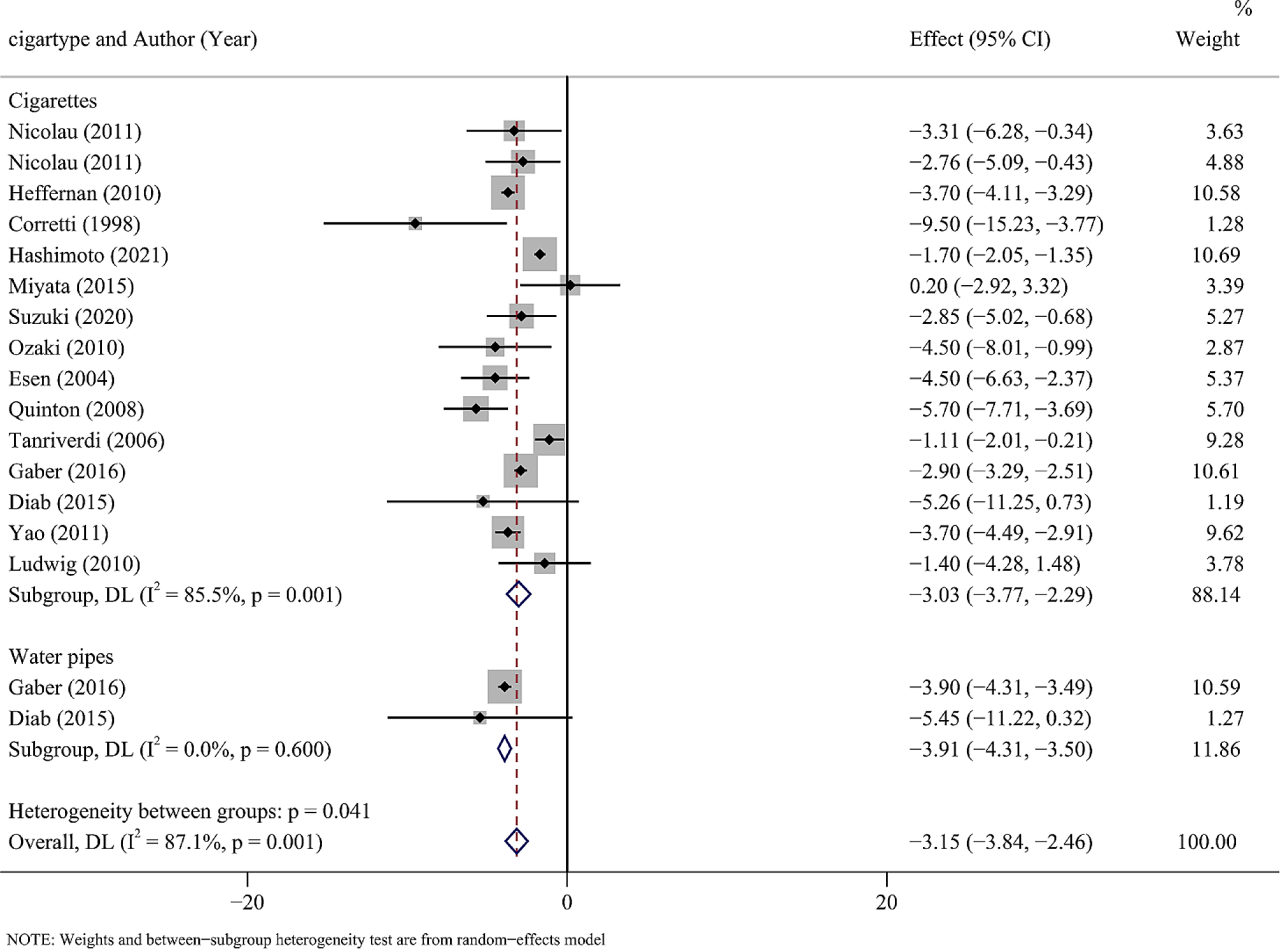
Forest plot of the tobacco type of the subgroup analysis

In terms of tobacco type, the results showed that both conventional tobacco and hookah reduced FMD values, possibly affecting their endothelial function (Cigarettes: *P* = 0.001, *I*^2^ = 85.5%, P_I2_ = 0.001; Water pipes: *P* = 0.001, *I*^2^ = 85.5%, P_I2_ = 0.600) (Fig. 3).

### Publication bias

Finally, Egger’s test was used to evaluate for publication bias, and the results revealed the absence of publication bias in our study (*P* = 0.429) (Additional file 2).

### Influence analysis

The findings of the study revealed that out of the 17 studies included, none of the remaining 16 studies exhibited statistically significant pooled results when any one study was excluded. This consistency with the original pooled effect size of -3.15 (95% CI: -3.84, -2.46) suggests that the results of the meta-analysis were robust and stable (Additional file 3).

## Discussion

Tobacco use is an important risk factor for CVDs, including hypertension, atherosclerosis, and stroke. One of the mechanisms by which smoking contributes to CVD is by altering endothelial function in blood vessels, which is assessed by FMD. An analysis of the 14 articles included in our meta-analysis suggested that smoking can reduce FMD and impair vascular endothelial function; notably, most of the studies were of good quality, with a quality assessment score of ≥ 7. Additionally, no heterogeneous changes in cigar type, pregnancy status, geography, or health status were identified in the subgroup analysis, suggesting the presence of other potential unknown causes.

Impaired FMD is well-known as a predictor of CVD, and smoking is known to decrease FMD in both smokers and nonsmokers. In the study by Celermajer et al. [23], Among smokers, the FMD values are notably reduced by 24% in comparison to non-smoking counterparts, with this detrimental effect persisting beyond four weeks post-smoking cessation. This observation implies a potential deleterious impact of smoking on vascular endothelial function, culminating in compromised vasodilatory capacity. The observed decrement in vasodilation is plausibly linked to the oxidative stress, inflammatory responses, and endothelial cell injury instigated by smoking, all of which are well-established contributors to the pathogenesis of atherosclerosis. The manifestation of FMD alterations is subject to variability influenced by lifestyle choices. In particular, the presence of diabetes mellitus may accentuate the adverse effects on FMD [24], whereas in other scenarios, the impact of smoking parallels that of diabetes [25–27]. Many studies have reported that smoking decreases FMD and impairs endothelial function, regardless of pregnancy status. For example, one study reported that smoking acutely decreases FMD in both pregnant and nonpregnant women, indicating that the deleterious effects of smoking on endothelial function are not limited to pregnant women [28]. Subgroup analysis of the study revealed that pregnant and nonpregnant populations did not represent one source of heterogeneity. Further, the deleterious effects of cigarette smoking on endothelial function and FMD are not limited to adults. Several studies have reported that exposure to passive smoke significantly reduces FMD, which further induces endothelial dysfunction, in healthy young adults [29, 30]. Exposure to parental smoking was found to be associated with reduced vascular function in both young children and adolescents, which was particularly evident in those whose parents had smoked during pregnancy [31]. These findings underscore the importance of protecting children and adolescents from exposure to tobacco smoke.

The association between smoking exposure and endothelial function evaluated using FMD values has been investigated in several studies. In a study conducted in Asia, the effect of cigarette smoking on FMD was studied in 71 Japanese men [32]. They found that smokers had a significantly lower FMD than nonsmokers. These studies suggest that smoking is associated with impaired endothelial function through FMD measurement conducted worldwide. Moreover, our results indicate that the presence or absence of Asian populations was not a source of heterogeneity. These investigations collectively illuminate a pervasive correlation between tobacco smoking and compromised endothelial function, as evidenced by assessments of Flow-Mediated Dilation (FMD). Nevertheless, the exactitude of FMD as a biomarker for endothelial dysfunction merits further elucidation. Despite its status as a prevalent non-invasive diagnostic instrument, the uniformity and dependability of FMD assessments may be contingent upon a spectrum of determinants, ranging from lifestyle factors to genetic underpinnings and environmental exposures.

Diabetes and obesity are known to independently contribute to a decline in flow-mediated dilation (FMD) through the augmentation of oxidative stress and the increased release of inflammatory cytokines from adipose tissue, such as tumor necrosis factor-alpha (TNF-α) and C-reactive protein (CRP) [33]. The chronic low-grade inflammatory state and insulin resistance associated with metabolic syndrome, including hypertension, further exacerbate the reduction in FMD. Studies have demonstrated that, compared to a control group, patients with hypertension exhibit significantly lower FMD values, and the migratory and proliferative functions of endothelial progenitor cells (EPCs) are markedly diminished (*P* < 0.05). Additionally, a strong positive correlation exists between the migratory and proliferative capacities of circulating EPCs and FMD (migration: *r* = 0.56, proliferation: *r* = 0.41, *P* < 0.05) [34]. In individuals with type 2 diabetes, chronic smokers have been found to have impaired microvascular reactivity, with a significant reduction in flow-mediated microvascular dilation response compared to non-smokers [35]. The deleterious effects of smoking on cardiovascular health that lead to endothelial dysfunction and reduced FMD are well-established, regardless of the presence of an underlying disease. In the current study, the patient characteristics were confined to either a healthy cohort or healthy pregnant women, thereby eliminating confounding factors that could distort the imaging outcomes. As a result, the findings are expected to be robust. Various studies on smoking cessation interventions have reported significant improvements in vascular endothelial function in successful quitters, regardless of the intervention, medication [36–39] or education for smoking cessation [39–41]. Furthermore, Schroeter et al. revealed that smoking-induced endothelial dysfunction is dose-dependent and reversible upon cessation of smoking [42]. Given that pregnancy status, Asian ethnicity, or health status did not affect heterogeneity, the existence of other unknown potential causes was considered, such as genotype or other environmental pollutant exposures. A previous study investigated the effect of a genetic variation in the promoter region of interleukin-6 (IL-6) gene on endothelial function in healthy volunteers. The findings of that study revealed that individuals carrying the C allele of the IL-6–174G > C polymorphism had impaired endothelial function compared to those carrying the GG genotype [43]. This finding highlights the potential role of the IL-6 gene in the CVD development via its endothelial function effects.

## Conclusions

The results of this study revealed that smoking alone is a major contributor to endothelial dysfunction and FMD impairment, regardless of pregnancy status, Asian ethnicity, and health status. Other possible causes of endothelial dysfunction and FMD impairment may include genotype variations.

### Electronic supplementary material

Below is the link to the electronic supplementary material.

Supplementary Material 1

Supplementary Material 2

Supplementary Material 3

## Data Availability

All data generated or analysed during this study are included in this published article and its supplementary information files.

## Abbreviations

FMD: Flow-mediated dilation
CVDs: Cardiovascular diseases
NO: Nitric oxide
NOS: Newcastle–Ottawa Scale
IL-6: Interleukin-6
