# Supplementary material for: The association between smoking exposure and endothelial function evaluated using flow-mediated dilation values: a meta-analysis
Source: BMC Cardiovasc Disord. 2024 Jun 5;24:292. doi: 10.1186/s12872-024-03915-x (PMC11151634; doi:10.1186/s12872-024-03915-x)
Supplement: Supplementary file 1 — Supplementary Material 1 [file 12872_2024_3915_MOESM1_ESM.docx]

**Additional file 1. Full Search Strategy**

**Date: Null-2023.02.21**

**PubMed**

#1 "endothelial function"[Title/Abstract] OR "endothelium function"[Title/Abstract]

#2 “flow-mediated dilation test”[Title/Abstract] OR “flow mediated dilat*”[Title/Abstract] OR “flow mediated vasodilat*”[Title/Abstract] OR “flow mediated vasoactivit*”[Title/Abstract] OR “FMD”[Title/Abstract] OR “flow-mediated dilat*”[Title/Abstract] OR “flow-mediated vasodilat*” [Title/Abstract] OR “flow-mediated vasoactivit*”[Title/Abstract]

#3 “smoking”[MeSH Terms] OR “smoking”[Title/Abstract] OR “electronic nicotine delivery systems”[MeSH Terms] OR “electronic nicotine delivery systems”[Title/Abstract] OR “Tobacco”[MeSH Terms] OR “Tobacco”[Title/Abstract] OR “cigarette”[Title/Abstract] OR “cigar*”[Title/Abstract] OR “electronic cigarette”[Title/Abstract] OR “e-cig”[Title/Abstract] OR “e-cigarette”[Title/Abstract]

#4 #1 AND #2 AND #3

**Embase**

#1 'endothelial function'/exp OR 'endothelial function':ab,ti OR 'endothelium function'/exp OR 'endothelium function':ab,ti

#2 'flow-mediated dilation test'/exp OR 'flow-mediated dilation test':ab,ti

#3 ‘flow mediated dilat*’:ab,ti OR ‘flow mediated vasodilat*’:ab,ti OR ‘flow mediated vasoactivit*’:ab,ti OR ‘FMD’:ab,ti OR ‘flow-mediated dilat*’:ab,ti OR ‘flow-mediated vasodilat*’:ab,ti OR ‘flow-mediated vasoactivit*’:ab,ti

#4 #2 OR #3

#5 ‘cigarette’/exp OR ‘cigarette’:ab,ti OR ‘smoking’/exp OR ‘smoking’:ab,ti OR ‘Tobacco’/exp OR ‘Tobacco’:ab,ti

#6 ‘electronic cigarette’/exp OR ‘electronic cigarette’:ab,ti OR ‘e-cig’:ab,ti OR ‘e-cigarette’:ab,ti OR ‘electronic nicotine delivery systems’:ab,ti OR ‘cigar*’:ab,ti

#7 #5 OR #6

#8 #1 AND #4 AND #7

**Web of Science**

(TI=("endothelial function" OR "endothelium function") OR AB=("endothelial function" OR "endothelium function")) AND (TI=(“smoking” OR “electronic nicotine delivery systems” OR “Tobacco” OR “cigarette” OR “cigar*” OR “electronic cigarette” OR “e-cig” OR “e-cigarette”) OR AB=(“smoking” OR “electronic nicotine delivery systems” OR “Tobacco” OR “cigarette” OR “cigar*” OR “electronic cigarette” OR “e-cig” OR “e-cigarette”)) AND (TI=(“flow-mediated dilation test” OR “flow mediated dilat*” OR “flow mediated vasodilat*” OR “flow mediated vasoactivit*” OR “FMD” OR “flow-mediated dilat*” OR “flow-mediated vasodilat*” OR “flow-mediated vasoactivit*”) OR AB=(“flow-mediated dilation test” OR “flow mediated dilat*” OR “flow mediated vasodilat*” OR “flow mediated vasoactivit*” OR “FMD” OR “flow-mediated dilat*” OR “flow-mediated vasodilat*” OR “flow-mediated vasoactivit*”))

**Cochrane Library**

#1 ("endothelial function":ti,ab OR "endothelium function":ab,ti)

#2 (“flow-mediated dilation test”:ab,ti OR “flow mediated dilat*”:ab,ti OR “flow mediated vasodilat*”:ab,ti OR “flow mediated vasoactivit*”:ab,ti OR “FMD”:ab,ti OR “flow-mediated dilat*”:ab,ti OR “flow-mediated vasodilat*”:ab,ti OR “flow-mediated vasoactivit*”:ab,ti)

#3 ([mh “cigarette”] OR “cigarette”:ab,ti OR [mh “smoking”] OR “smoking”:ab,ti OR [mh “Tobacco”] OR “Tobacco”:ab,ti)

#4 ([mh “electronic nicotine delivery systems”] OR “electronic nicotine delivery systems”:ab,ti OR “electronic cigarette”:ab,ti OR “e-cig”:ab,ti OR “e-cigarette”:ab,ti OR “cigar*”:ab,ti)

#5 #3 OR #4

#6 #1 AND #2 AND #5
