# Supplementary figures and images for: The association between smoking exposure and endothelial function evaluated using flow-mediated dilation values: a meta-analysis
Source: BMC Cardiovasc Disord. 2024 Jun 5;24:292. doi: 10.1186/s12872-024-03915-x (PMC11151634; doi:10.1186/s12872-024-03915-x)

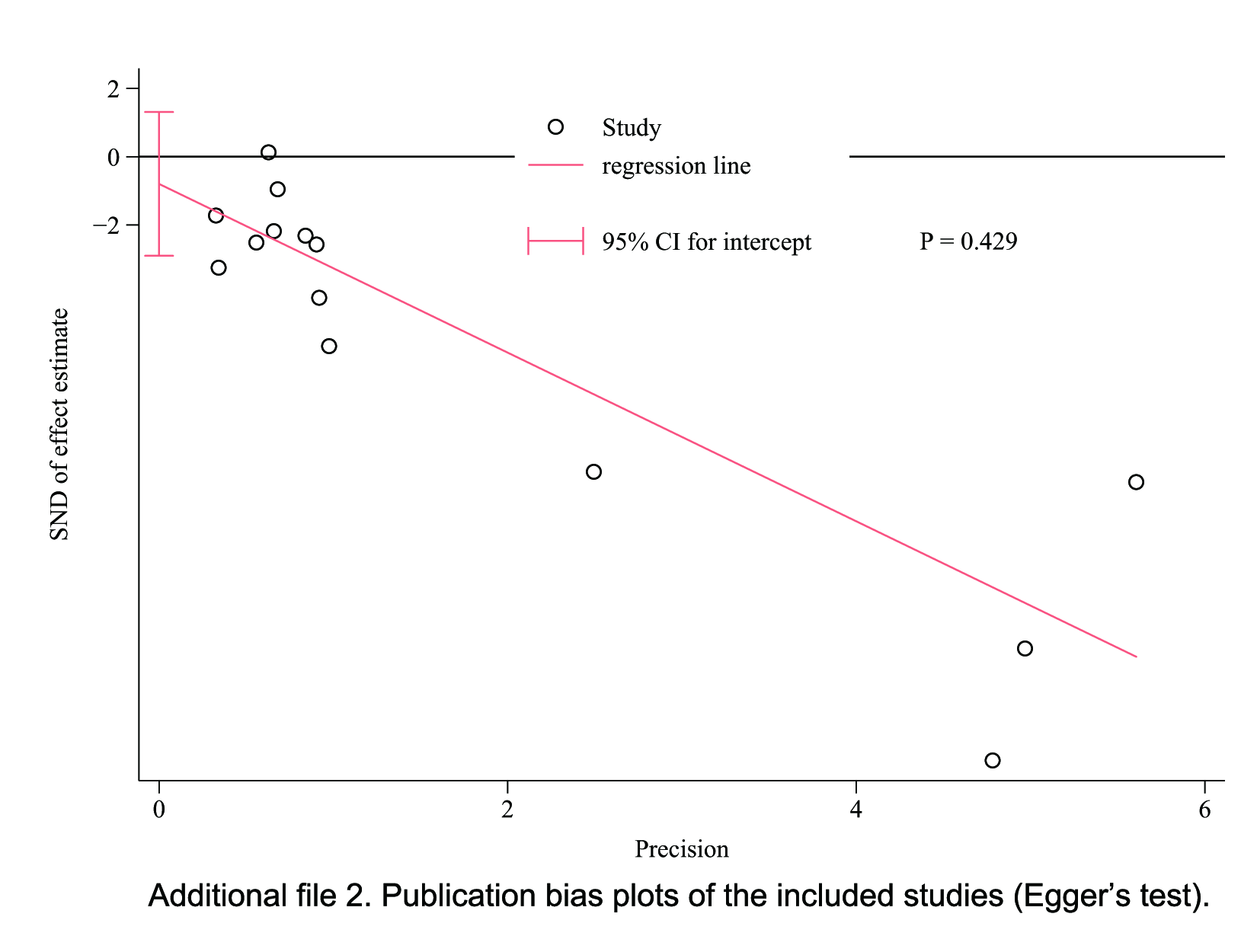

Supplement: Supplementary file 2 — Supplementary Material 2 [file 12872_2024_3915_MOESM2_ESM.tif]

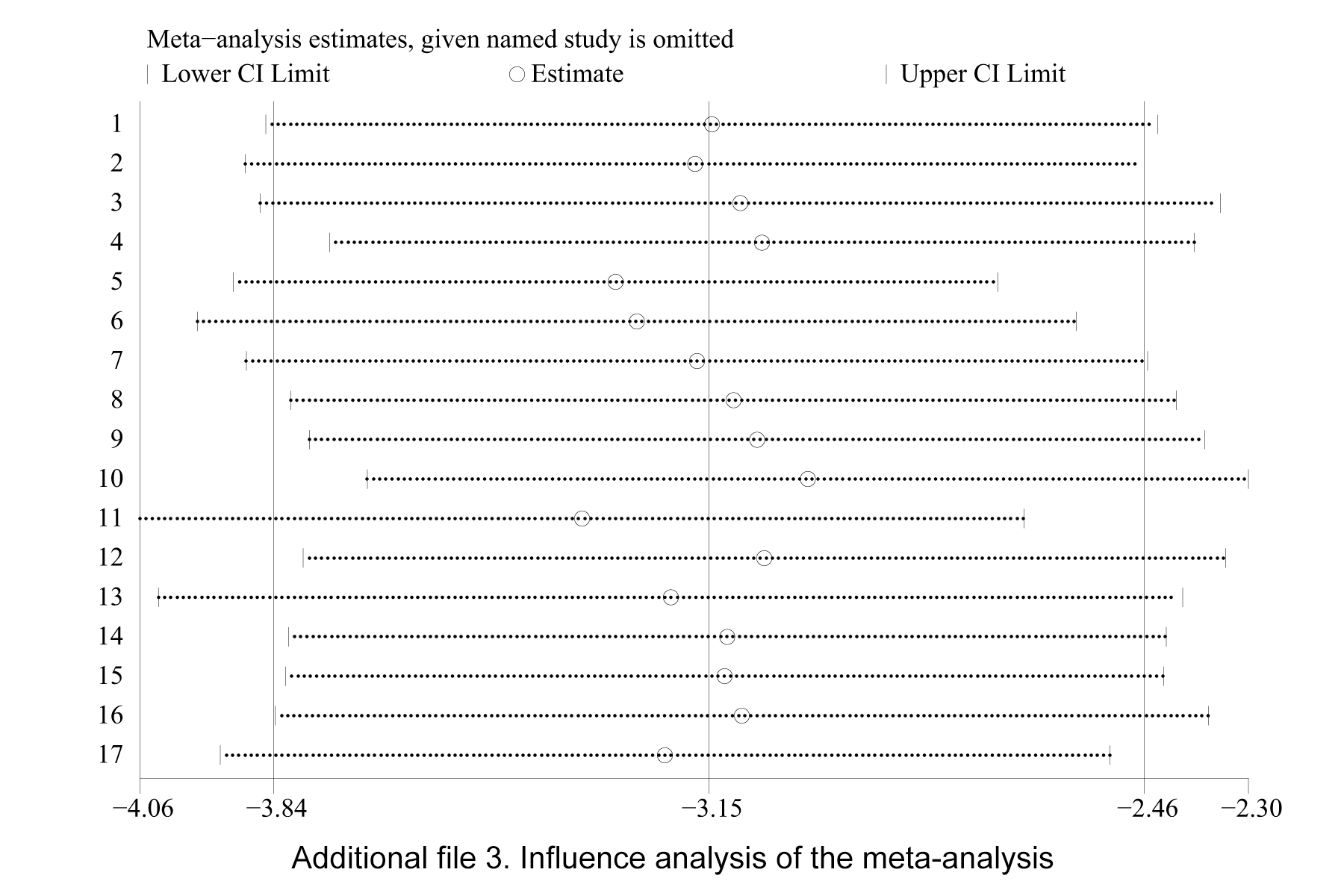

Supplement: Supplementary file 3 — Supplementary Material 3 [file 12872_2024_3915_MOESM3_ESM.tif]
